# Arctic Vortex changes alter the sources and isotopic values of precipitation in northeastern US

**DOI:** 10.1038/srep22647

**Published:** 2016-03-14

**Authors:** Tamir Puntsag, Myron J. Mitchell, John L. Campbell, Eric S. Klein, Gene E. Likens, Jeffrey M. Welker

**Affiliations:** 1 SUNY-ESF, Syracuse, NY 13210, USA; 2 US Forest Service, Northern Research Station, Durham, NH 03824, USA; 3 University of Alaska Anchorage, Biological Sciences Department, AK 99508, USA; 4 Cary Institute of Ecosystem Studies, Millbrook, NY 12545, USA; 5 University of Connecticut, Department of Ecology and Evolutionary Biology, Storrs, CT 06269, USA

## Abstract

Altered atmospheric circulation, reductions in Arctic sea ice, ocean warming, and changes in evaporation and transpiration are driving changes in the global hydrologic cycle. Precipitation isotopic (δ^18^O and δ^2^H) measurements can help provide a mechanistic understanding of hydrologic change at global and regional scales. To study the changing water cycle in the northeastern US, we examined the longest (1968–2010) record of precipitation isotope values, collected at the Hubbard Brook Experimental Forest in New Hampshire, US (43^o^56′N, 71^o^45′W). We found a significant reduction in δ^18^O and δ^2^H values over the 43-year record, coupled with a significant increase in *d-excess* values. This gradual reduction in δ^18^O and δ^2^H values unexpectedly occurred during a period of regional warming. We provide evidence that these changes are governed by the interactions among the Atlantic Multidecadal Oscillation, loss of Arctic sea ice, the fluctuating jet stream, and regular incursions of polar air into the northeastern US.

The global water cycle is exhibiting dramatic changes as surface air and sea surface temperatures have increased, perennial sea-ice has decreased, droughts have become more extreme, severe flooding due to sea level rise and protracted winter storms have become more common, and precipitation variability has increased (e.g., heavy downpours are likely to occur more frequently in the Northeast)^1^,^2^. For example, in the winter of 2013–2014 there was a prolonged meridional flow across North America that led to an abnormally cold and snowy winter in the eastern US, a prolonged drought in the western US, and unusually warm winter temperatures across Alaska^2^. The mechanisms controlling these changes are complex and include altered atmospheric circulation, reductions in Arctic sea ice, ocean warming, and changes in evaporation on water bodies (lakes, seas and oceans) and transpiration^3^. Because isotope (δ^18^O and δ^2^H values) ratios in precipitation have been shown to respond to climate oscillations and other abiotic influences, they provide a powerful tool to help understand the underlying processes affecting hydrologic changes at global and regional scales^4^,^5^,^6^,^7^,^8^.

Condensation and evaporation influence water isotopic (δ^18^O and δ^2^H) values at both global^9^ and continental-scales, creating predictable patterns of precipitation geochemistry^4^,^10^,^11^. These water isotopic fractionation processes are influenced by various physical factors, including temperature^12^,^13^. However, it has been shown that other critical factors including moisture sources^14^, air mass trajectory^5^, seasonality^14^, and teleconnections [e.g., Pacific North American (PNA) and El Niño Southern Oscillation (ENSO)], collectively influence the water cycle and isotopologues of preciptation^8^,^12^. Climatic influences and storm track effects on precipitation isotopes have been primarily examined for western North America^4^,^5^,^11^ or over short-time periods in the eastern US^14^. A long-term perspective is needed, however, to evaluate fluctuations in climate phases and shifts in storm track patterns. We expect that climate change indices, including the Atlantic Multidecadal Oscillation (AMO), the North Atlantic Oscillation (NAO), and the Arctic Oscillation (AO), may show linkages to changes in precipitation isotopes^15^. In our study we show that *d-excess* (δ^2^H-8 × δ^18^O) values provide new information on water sources affected by evaporation or other non-equilibrium phase changes such as diffusion and dissociation reactions^10^.

To examine long-term trends in the hydrologic cycle of the northeastern US, we developed the longest (1968–2010)^4^,^8^,^12^,^16^ continuous record of precipitation isotopes available, using archived samples collected at the Hubbard Brook Experimental Forest (HBEF). A substantial amount of past research incorporates natural paleoclimatic records (e.g., ice cores, lake sediments, tree rings, limestone caves, and groundwater) that preserve the isotopic composition of water and are useful in evaluating the relationships between climatic variables and precipitation δ^18^O values. However, relatively little research has investigated modern relationships between stable isotopologues of water and climatic patterns over extended periods (i.e., decades). Our study provides new information on the modern record of precipitation and how isotopic values (δ^18^O and δ^2^H) and *d-excess* reflect the sources of precipitation and how they have changed in response to climatic changes over the past 43 years.

## Results

### Long-term trends

**Figure 1:**
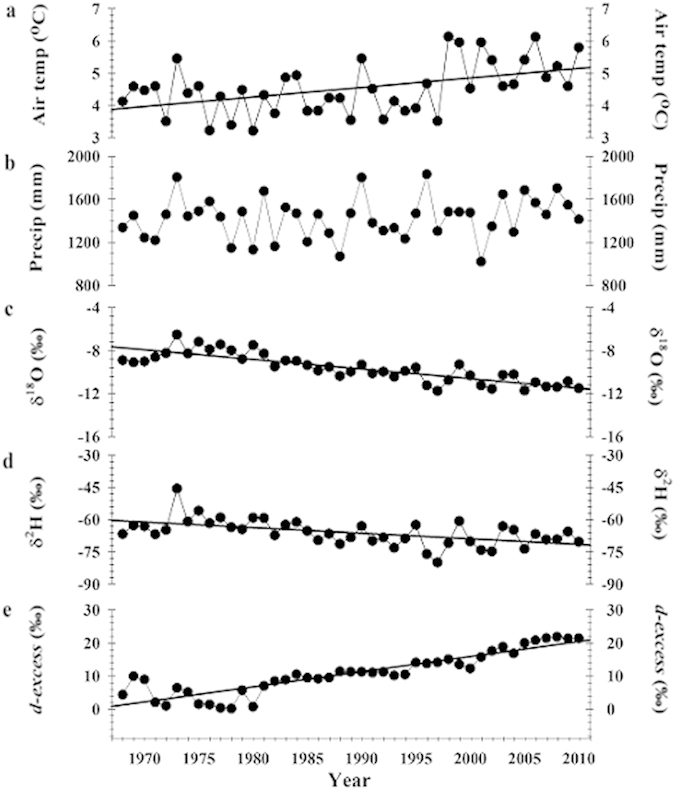
Long term annual mean values and trends in climate and precipitation isotopes at northeastern US site. (**a–e**) Results indicate a (**a**) significant increasing trend in surface air temperature (slope = 0.03 °C yr^−1^, P = 0.0017); (**b**) no significant trend in precipitation amount (slope = 2.71 mm yr^−1^, P = 0.25); **(c)** significant declines in δ^18^O (slope = −0.089‰ yr^−1^, P < 0.0001) and (**d**) δ^2^H (slope = −0.259‰ yr^−1^, P = 0.0002); and (**e**) and a significant increase in *d-excess* (slope = 0.46‰ yr^−1^, P < 0.0001).

We found significant positive trends in mean annual *d-excess* values (P < 0.0001) and surface air temperatures (P = 0.005), whereas δ^18^O (P < 0.0001) and δ^2^H (P = 0.0003) values showed significant declines over the 43-year period (Fig. 1a–e). Average annual surface air temperatures ranged from a minimum of 3.2 °C in 1980 to a maximum of 6.1 °C in 1998 over the 43 years (Fig. 1a). The precipitation amounts ranged from a minimum of 100 cm in 2001 to a maximum of 180 cm in 1973 (Fig. 1b). Annual isotopic values weighted by monthly precipitation amounts for δ^18^O ranged from a relatively high value of −6.5‰ in 1973 (the wettest year^16^) to a low value of −12‰ in 1997 (Fig. 1c) while δ^2^H values for these same years were −46‰ and −80‰ respectively (Fig. 1d). Annual *d-excess* values ranged from a low value of 0.2‰ in 1978 to a high value of 22‰ in 2008 (Fig. 1e). Based on the significant linear relationship (P = 0.02), a 1 °C change in temperature resulted in a −0.61‰ change in the δ^18^O values from 1968–2010, whereas the *d-excess* and surface air temperature values were positively related (P < 0.0001) with a slope of 4.6‰/1 °C. Over the 43-year record, the annual average *d-excess* values increased from ~4‰–21‰, weighted annual δ^18^O values declined from −8.9‰ to −11.5‰, and δ^2^H values declined from −66.7‰ to −70.3‰ (Fig. 1c–e).

**Figure 2:**
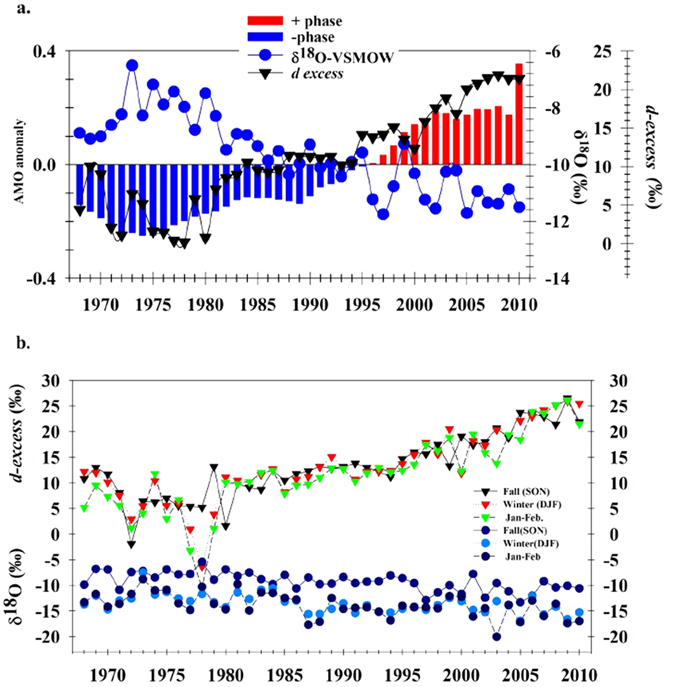
Response of HBEF precipitation δ^18^O and *d-excess* values during the study period to AMO phase changes. (**a,b**) Average, de-trended AMO anomalies (^o^C) from the Kaplan sea surface temperature V2 from http://www.esrl.noaa.gov/psd/data/timeseriesimeseries/AMO), δ^18^O = −6.78AMO-9.90, r^2^ = 0.7 and *d-excess* = 35.3AMO + 12.4, r^2^ = 0.83 (**a**) and comparison of fall precipitation δ^18^O (blue dots) and *d-excess* value (black triangle), winter precipitation δ^18^O (light blue dots) and *d-excess* value (red triangle) with a precipitation δ^18^O of January-February (dark blue) and *d-excess* value (green triangle) over the 1968–2010 study period (**b**).

The progressive reduction in δ^18^O and δ^2^H values during our study period differs from some global and regional results, which have shown that δ^18^O and δ^2^H values of precipitation increase with mean annual surface air temperatures^9^,^10^. However, temperature generally only accounts for approximately 50–60% of the variance^4^,^9^,^13^ in precipitation isotopic values, indicating that other processes, such as climate oscillations, moisture sources and changes in atmospheric circulation are also important controlling mechanisms^4^,^5^,^6^,^8^,^17^. To further explore the influence of climate change on the isotopic composition of precipitation, we examined the correspondence between climate oscillations and the δ^18^O and *d-excess* values for precipitation at the HBEF over the last four decades, including the AMO^18^,^19^,^20^,^21^, AO^22^, NAO^23^, and PNA^5^. The AMO is a North Atlantic Ocean current (0–70^o^N) with decadal modes that affect sea surface temperature variability; the AO and NAO reflect sea level pressure anomalies poleward of 20^o^N and opposing variations of barometric pressure near Iceland and the Azores; the PNA reflects an atmospheric large-scale wave pattern featuring a sequence of tropospheric high and low pressure anomalies stretching from the subtropical west pacific to the east coast of North America. We found that only the AMO was significantly (P < 0.0001) related to HBEF precipitation δ^18^O, δ^2^H and *d-excess* values (Supplementary Table S1 and Fig. S2). A multi-decadal fluctuation in the North Atlantic, in which sea surface temperature exhibited a positive phase from 1968–1994 and a negative phase from 1995–2010 with a temperature range of ~0.4 °C (Fig. 2a). All oscillations were associated with variation in the direction and strength of the prevailing circulation and storm track affecting moisture sources, temperature and precipitation amounts that were most strongly expressed in winter, but affected climate throughout the year.

### Influential factors on precipitation isotopes

We performed stepwise multiple-regression (mixed option) for both δ^18^O and *d-excess* values as dependent variables, and mean annual temperature (°C), precipitation amount (mm), AMO, NAO, PNA, and AO indices as independent variables as a means of resolving the multitude of potential factors influencing our isotopic values in precipitation. We found that 84% of the variation in *d-excess* (adjusted r^2^ = 0.84; RMSE = 2.5) was explained by AMO, precipitation amount, and AO, as described by the following equation (1) (Fig. S3b).


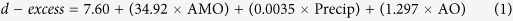


For δ^18^O values, AMO index and mean annual temperature explained 70% of the variation of δ^18^O values (adjusted r^2^ = 0.70; RMSE = 0.74) as described by the following equation (2) (Fig. S3a).


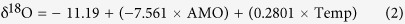


These analyses support our hypothesis that the AMO played a dominant role in affecting HBEF precipitation *d-excess* values, with the AO and local precipitation amounts having a significant, but secondary effect based on the smaller, t-test P values (below α_e_ = 0.15) in the stepwise model. Similarly for the δ^18^O values, the AMO values also had the strongest influence, with local mean annual temperature values having a secondary, but significantly important contribution to the variance in δ^18^O values. The AMO index does not reflect a specific climate pattern (jet stream flow trait), but rather reflects ocean warming and any secondary consequences of that warming at large spatial scales^18^,^24^. During our study period, there was an increasing trend in AMO values (as measured by the AMO anomaly based on decadal changes in sea surface temperature) from −0.25 to +0.35, which corresponded with a positive trend in *d-excess* values from ~0 to 22‰ and decrease in the δ18O values from ~−7 to -12‰ (Fig. 2a).

The progressive increase in *d-excess* values indicates that precipitation moisture sources during our study (1968–2010) were increasingly from colder northern regions; an observation similar to a much shorter-term study on the role of Arctic moisture sources on the precipitation isotope geochemistry in the northeastern US^14^,^25^. This previous study^14^ used back trajectories of daily precipitation isotope values between 1999–2001 to show that this region receives moisture from the Arctic ~15% of the time, with *d-excess* values of ~19‰, and that this moisture source typically occurs during the fall and winter. Thus, increases in the frequency of Arctic moisture sources have the potential to influence the average annual isotopic values of sites in the northeastern US. When surface air temperatures in these northern regions are near the freezing point of water, the ratios of the ice/vapor fractionation factor are higher for δ^2^H compared to δ^18^O due to the greater fractionation of hydrogen isotopes under these conditions^17^,^26^. Hence, greater *d-excess* values may reflect moisture sources that are derived from colder and drier climates compared to moisture sources associated with more humid and warmer climates^17^,^26^,^27^. Also, during positive AMO conditions, the North Atlantic jet stream and storm tracks shift southward, leading to incursions of air from higher latitudes across the eastern US and northern Europe^24^,^28^. Often *d-excess* changes are indicative of shifts in moisture sources, with low *d-excess* values associated with substantial evaporation in coastal zones, and high values associated with moisture sources over terrestrial regions characterized by substantial amounts of water recycled from plant transpiration^29^.

## Discussion

### Mechanisms controlling precipitation isotope trends

Complex changes in climate and synoptic weather patterns, with various climatic controls predicted by numeric models^24^,^30^, are the apparent mechanisms controlling the long-term changes in isotopic values of northeastern US precipitation. Arctic amplifications (1990–2010) and North Atlantic regional air temperature increases corresponded to a concomitant warming of the North Atlantic Ocean (0.031 °C per decade during the period of 1900–1999^23^ and ~0.4 °C per decade from 1990–2008^19^,^20^). Additionally, sea ice extent in the Arctic has decreased in concert with a general weakening of the polar vortex, leading to weakened west-to-east winds, and ultimately a more north/south meandering in the jet steam, allowing cold air excursions to become more frequent in the eastern US^19^,^24^,^31^. The markedly warmer Arctic, with decreased cover of sea ice in fall and early winter, has led to larger heat fluxes from the ocean to the polar stratosphere and a weakening of Arctic vortex and negative AO values, especially in mid-winter (January-February)^32^. This weakened polar vortex has resulted in changing weather patterns, especially at mid-latitudes. We propose that these changes in synoptic weather patterns (storm tracks) have resulted in the delivery of more frequent precipitation events from the north, which has led to a decline in δ^18^O and δ^2^H values and an increase in *d-excess* values for precipitation at the HBEF during a period of regional warming. These results are in contrast to most previous interpretations of declining isotopic values of precipitation associated with climate, which suggested that the declining values are a function of cooling atmospheric temperatures without incorporating the influence of moisture source changes^12^. These trends in δ^18^O, δ^2^H and *d-excess* values were also apparent during a shorter sampling period (1989–2003) at a site in northern Vermont^8^.

Fall sea ice in the Arctic Ocean has declined at a rate of 12.4%/decade since 1979, leading to progressively larger Arctic Ocean heat fluxes that impact the jet stream in a weaker zonal jet with larger meanders and more persistent extreme weather^24^,^31^. During this same period, δ^2^H and δ^18^O values at the HBEF have declined, while *d-excess* values have increased (Fig. 2b), suggesting a shift to an increased proportion of northern moisture sources. If these changes in the jet stream, which allow more air from the Arctic to cascade south into the northeastern US, were associated with a decrease in fall Arctic Ocean sea ice extent, it would be expected that the greatest isotopic changes in precipitation should occur in the fall and winter. This seasonal expectation is consistent with our findings at the HBEF, as most of the long-term annual changes in precipitation isotopic values were in the fall and winter periods (Fig. 2b). Corroborating evidence for a seasonal, as well as a moisture source shift is provided by marine aerosol studies^33^. For instance, from 1967–1994, trends in δ^34^S values in precipitation were positively correlated with marine SO_4_^2−^ concentrations,; winter (6%) marine contributions were higher than summer (3%) contributions^33^. Also, Ottawa in Ontario, Canada (45^o^32′N, 75^o^60′W) which is farther to the west and an inland site also shows a generally increasing trend in the average annual δ^18^O values of precipitation, but the trend was not statistically significant for 1970–2007^34^. The Ottawa site mean temperature was (6.1 °C) and was about 1.7 °C warmer than the HBEF site (4.4 °C) during this study; however this research showed a similar positive relationship between monthly δ^18^O in precipitation and temperature. It should be noted that at both sites the seasonal temperature ranges were much greater than long -term temperature ranges, making it challenging to evaluate the effects of long-term changes in temperature on precipitation isotopic values. Therefore, we suggest that the two studies are consistent with respect to seasonal temperature effects with the summer having the highest δ^18^O values and the winter the lowest. These observations further support our interpretation of how interrelated changes in synoptic climatology are driving long-term trends δ^18^O, δ^2^H and *d-excess* values in northeastern precipitation.

**Figure 3:**
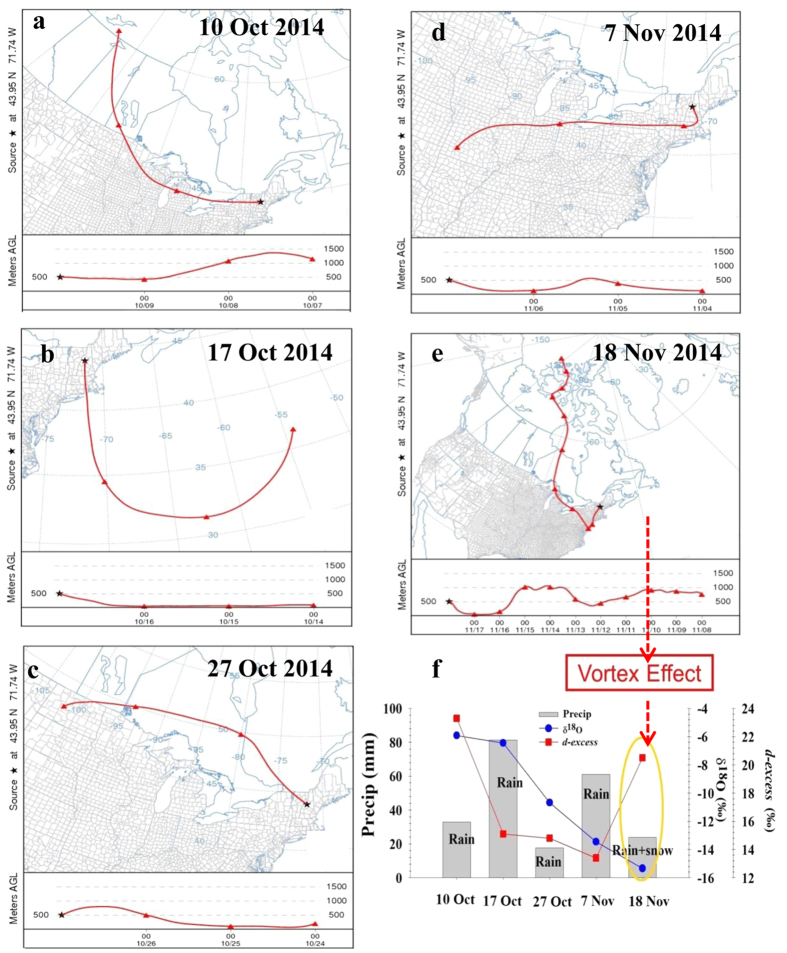
Polar vortex event in fall of 2014. Weekly sample results are shown in graph (**f**) with air parcel 72-hour back trajectories (**a**–**e**). The date on x axis of the graph (**f**) associated with selected trajectories are presented in a-e. The maps: output of the public website service software HYSPLIT^40^, https://www.ready.noaa.gov/HYSPLIT_traj.php and http://journals.ametsoc.org/doi/abs/10.1175/BAMS-D-14-00110.1.

The large changes in precipitation isotopes during the fall further suggest that the AMO captures important factors that lead to changes in large, spatial scale weather and moisture sources, including the generation of storms in the northeastern US derived from relatively cold and dry moisture sources (i.e., the Arctic). Air parcel back trajectories demonstrate how colder northern-sourced moisture can influence the isotopic patterns apparent in the long-term record (Fig. 3a–e). In November 2014 a polar vortex^35^ extended into the eastern US during a positive phase of the AMO. The result of this event, which ended ~November 18, was mixed rain-snow with comparatively low δ^18^O (−16‰) and high *d-excess* values (20‰). We also did isotopic analyses on precipitation events before the November event (Fig. 3f). A progressive increase in the frequency of these largely seasonal incursions of colder northern air would result in the isotope patterns we observed in the long-term isotope record at the HBEF.

**Figure 4:**
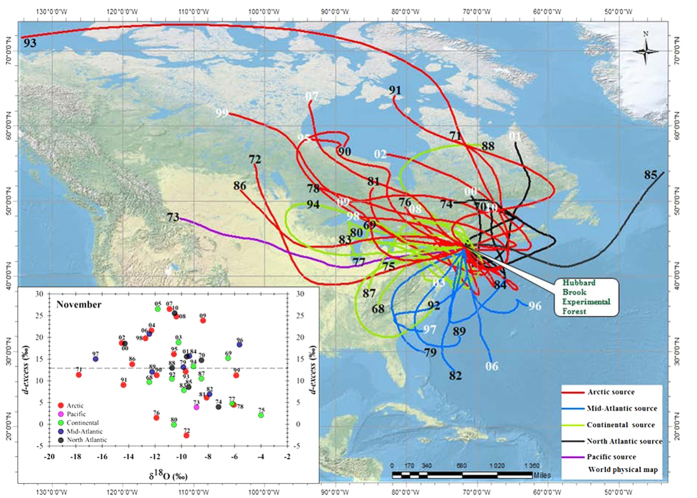
Air parcel back trajectories (main) and δ^18^O- *d-excess* values (inset) plot for November, 1968–2010. Five different colors indicate the 5 sources during the years associated with a negative AMO (black year numbers) and a positive AMO (white year numbers) phase. 40%, 26%, 16%, 16% 2% of trajectories show Arctic (red), Continental (green), North Atlantic (black), Mid-Atlantic (dark blue) and Pacific (purple) show, respectively. 57% of trajectories show northern sources: including Arctic and North Atlantic after the start of 1979 sea ice decline and 69% of trajectories show northern sources during positive AMO (start 1995) with higher *d-excess* values (>15‰). Figure created using ArcGIS 10.2.2.3552 (http://www.esri.com/software/arcgis/) with HYSPLIT model results^40^ http://journals.ametsoc.org/doi/abs/10.1175/BAMS-D-14-00110.1.

Additionally, temporal variations of air parcel back trajectories were estimated for the highest precipitation amounts for a day in November (last full month of fall) from 1968–2010 and compared with *d-excess* and δ^18^O values of precipitation (Fig. 4). These back-trajectory analyses were used for temporally constraining the spatial precipitation sources. We used the day with the highest daily precipitation as well as the day before and after the highest daily precipitation day for the November of each year. Precipitation amounts derived from these three daily air masses (72- h period) ranged from 24 to 73% of the total November precipitation for our study period.

This analysis indicates that the vast majority (11 of 16) of high *d-excess* values in precipitation were derived from northern sources, including the Arctic and North Atlantic, during the positive phase of AMO (Fig. 4). The highest (2007) and the lowest (1972) *d-excess* values of precipitation were, however, both associated with moisture sources from the Arctic. The isotopic and trajectory data suggest that the moisture sources during these two events in 2007 and 1972 were from: a) a humid (open Hudson Bay) region, with relatively high δ^18^O and low *d-excess* values and b) an arid region, with relatively low δ^18^O and high *d-excess* values of precipitation (Fig. 4). Conversely, continental sources tended to dominate during negative AMO phases.

**Figure 5:**
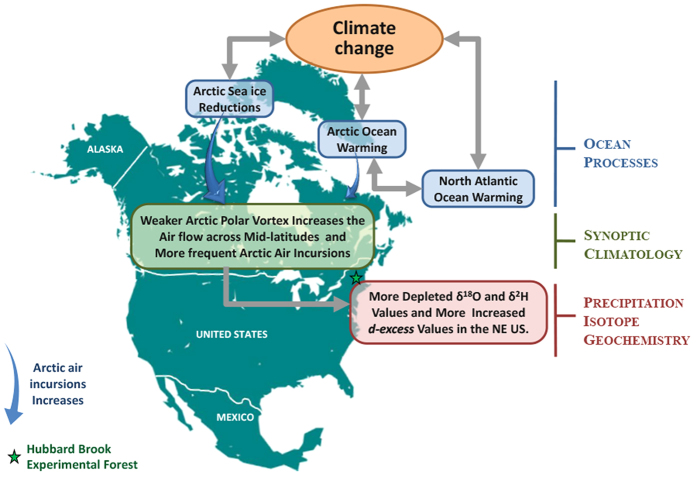
Conceptual diagram showing the direct and indirect effects of changes in ocean processes and synoptic climatology on the isotopic values of northeastern US precipitation. Climate change is linked with decreases in Arctic sea ice and increases in the surface temperatures of the Arctic and north Atlantic oceans. These changes are linked to a reduction of the strength of the Arctic polar vortex and resultant increase in air flow in the mid-latitudes and greater frequency of incursions of Arctic air in the northeastern US. Due to changes associated with isotopic fractionation processes and the sources of precipitation, the values of δ^18^O and δ^2^H have declined and *d-excess* values have increased. The map of North America (https://commons.wikimedia.org/wiki/File:Cartography_of_North_America.svg) is licensed under the Attribution-Share-Alike 3.0 Unported license. The license terms can be found on the following link: https://creativecommons.org/licenses/by-sa/3.0/. Figure created using Adobe Photoshop CS6 13.01×32.

We have established that δ^18^O and δ^2^H values in precipitation decreased while *d-excess* values increased over a 43-year period in the northeastern US and that these changes were linked with the Atlantic Ocean-Arctic amplification interactions. A broad conceptual overview of our results is provided in Fig. 5. A growing body of evidence suggests that the extreme cold that has occurred in the northeastern US in the fall and winter is a pattern we can expect to continue with increasing frequency as climate change progresses. This climatic pattern is due in part to Arctic warming which has been twice as rapid as that in the mid-latitudes. One result of this climatic shift is that the temperature differences between the Arctic and mid-latitudes are shrinking. These temperature patterns affect the polar vortex which is a sinistral swirling mass of cold air that spreads over the Arctic. The weakening of the Arctic and mid-latitude temperature differences leads to greater undulations of the polar vortex that causes larger excursions of cold air southward into the mid-latitudes, including the northeastern US. The isotopic trends found in our study will likely continue and possibly become stronger with the expected further weakening of the polar vortex^21^,^24^,^32^. In addition to understanding the factors that affect the modern isotopic values of precipitation, our results have important implications for the interpretation of hydrogen and oxygen isotopes in climate proxies^36^. For instance, interpretation of tree ring records of climate recorded in the ^18^O values of cellulose can now be considered in the context of moisture source shifts in the AMO, which is known to reflect multidecadal precipitation isotopic anomalies, and appears to modulate hurricane and drought frequency^18^.

Our results highlight the need to understand the influence of moisture sources and storm tracks, climate phases, sea ice, and land surface traits on isotopic values in regional precipitation and overall climatic patterns^4^,^5^,^6^. Additionally, our findings suggest that the isotope geochemistry of precipitation at the HBEF and northeastern US in general reflect increased North Atlantic sea surface temperatures and can be attributed in large part to increases in the proportion of Arctic precipitation sources associated with the decreasing extent of sea ice^37^. Hence the use of precipitation isotopes provides an additional tool for understanding changes in the northern regions including warmer temperatures and increased precipitation in the northeastern US. Such information should be incorporated into predictive models of regional and global climates. Evaluating these patterns is critical for understanding the complexities of global climate change and how the connections between marine and terrestrial systems influence the changing hydrologic cycle.

## Methods

### Site description

The Hubbard Brook Experimental Forest (HBEF) is located within the White Mountain National Forest of north central New Hampshire (43^o^56′N, 71^o^45′W), approximately 120 km northwest of the North Atlantic Ocean. The climate of the HBEF is humid continental with short, cool summers and long, cold winters^16^,^33^. During the fall and winter, as the colder polar air moves south, cyclonic disturbances periodically move up the east coast of the US providing an occasional source of maritime air^14^,^16^. The mean annual air temperature (measured between 1968 and 2010) was 4.8 °C, with a monthly mean maximum of 17.5 °C in July and a monthly mean minimum of −9.6 °C in January using data collected at the base of the biogeochemical reference watershed (W6). During our study the mean annual precipitation amount was 138 cm, with a monthly maximum of 12.8 cm in August and a monthly minimum of 8.7 cm in February. On average, precipitation at the HBEF is distributed equally throughout the year with approximately 30% falling as snow. A snowpack usually covers the ground from late December until mid-April; the average annual maximum depth of 72 cm (19 cm snow water equivalent) occurs in March^38^.

### Sample collection and handling

Precipitation samples at Rain Gauge 11 (RG11) near the base of W6 were collected weekly in a bulk-precipitation collector consisting of a 28-cm diameter polyethylene funnel attached to Tygon^®^ tubing leading to a 2-liter reservoir. The Tygon^®^ tubing was looped to create a vapor barrier that minimizes evaporation. Snow for chemical analysis was collected in plastic bags during the winter^16^. After chemical analysis of the major solutes, the remaining samples were stored in screw-top, sealed Nalgene^®^ bottles in the archive building, where temperatures are kept between 5 and 10 °C. For the current study, we used archived precipitation samples collected weekly from 1968, when samples were first archived, through 2010 (43 years total). After compositing the weekly samples by volume (to the nearest 0.1 ml) into a monthly sample, the samples were shipped to the Stable Isotope Laboratory at the University of Alaska in Anchorage. Event samples were collected between November 11 and 18, 2014, during a shift in the polar vortex. Isotopic analysis was performed using a Picarro Cavity Ring Down Spectrometer (Li-1115) fitted with an auto-sampler. Each sample was analyzed six times and reanalysis of the sample was done when the standard deviation of the six replicates was >0.3‰ for δ^18^O and 3‰ for δ^2^H, or when the internal standard for the run differed from the accepted value by >± 0.2‰ and 2‰ for δ^18^O and δ^2^H, respectively. Internal standards (USGS 45 and 46) and processed Anchorage tap water (with a known value) were used with each tray (50 samples) to account for any daily drift. All results are reported relative to Vienna Standard Mean Ocean Water (VSMOW) and were calibrated using IAEA VSMOW, Standard Light Antarctic Precipitation (SLAP), and Greenland Ice Sheet Precipitation (GISP) standards.

### Data and analysis

HBEF daily climate data (available at http:/hubbardbrook.org) from 1968–2010 were combined into monthly and annual averages. We used the NAO, PNA, AMO and AO indexes from the National Oceanic and Atmospheric Administration (NOAA) and National Climatic Data Center (Fig. S1). We calculated annual average temperatures and used ANOVA to evaluate differences by year. We used Kendall’s tau in SAS 9.1.3 for trend analysis of the monthly samples to evaluate how water isotope values and surface air temperature and precipitation values changed over time. Selections of climate variables were made using a multiple stepwise regression approach in JMP in 5.1.1software. For all δ^18^O and δ^2^H values at the HBEF we used weighted monthly precipitation to derive annual values (δ_weighted annual_) calculated as^9^:


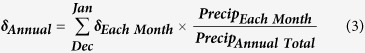


Using the NOAA’s Air Resources Laboratory (ARL) HYbrid Single-Particle Lagrangian Integrated Trajectory (HYSPLIT) model^39^, we calculated 72-h back trajectories for air masses at 500 m above ground level. The 500- m back trajectories show a more easterly component representing surface air drawn into an approaching frontal system. We used the National Center for Atmospheric research (NCAR) Reanalysis Project data set archived by ARL for meteorological data. The back trajectory end point is RG11 at the HBEF (43.950227N and -71.734612W). Back trajectories and precipitation source regions were evaluated for each year at our study site^14^.

### Sample integrity for water isotope analyses

The first objective of these determinations was to evaluate the potential influence of evaporation on water isotopic values of samples stored in the archives. A concern regarding the use of the archived samples for isotopic analyses is evaporation/condensation and vapor exchange with external air. The local Meteoric Water Line (MWL) values for the samples collected in our study were very close to that of the Global MWL as well as for a site in Ottawa, Canada (Fig. S4). Only 8% of the 516 samples had *d-excess* values that were less than 0‰, suggesting that our samples did not undergo significant secondary evaporation during storage, preserving their integrity and isotopic reliability^8^,^9^,^26^,^29^. We also compared our results with samples collected at similar times (i.e., within a few days) from 2006–2010 of bulk precipitation (Watershed3-RG4) near the location used in our long-term analyses. Mineral oil was added to the RG4 collectors to prevent evaporation in the field, and samples were stored in 20 mL glass vials that were completely filled with sample water and sealed with caps that contained plastic conical inserts to remove headspace and prevent evaporation. The isotopic values for the RG4 samples (n = 42) showed no substantial differences (<5% and −2‰ (δ^18^O), −8‰ (δ^2^H) heavier and 8‰ (*d-excess*) smaller) between measurements to those values from RG11 samples in the HBEF archive taken for the same month.

## Additional Information

**How to cite this article**: Puntsag, T. *et al*. Arctic Vortex changes alter the sources and isotopic values of precipitation in northeastern US. *Sci. Rep*. **6**, 22647; doi: 10.1038/srep22647 (2016).

## Supplementary Material

Supplementary Information
