# Supplementary material for: Arctic Vortex changes alter the sources and isotopic values of precipitation in northeastern US
Source: Sci Rep. 2016 Mar 14;6:22647. doi: 10.1038/srep22647 (PMC4789600; doi:10.1038/srep22647)
Supplement: Supplementary Information [file srep22647-s1.pdf]

# Arctic Vortex changes alter the sources and isotopic values of precipitation in northeastern US

Tamir Puntsag[1*], Myron J Mitchell[1], John L Campbell[2], Eric S Klein[3], Gene E Likens[4] and Jeffrey M Welker[3]

[1]SUNY-ESF, Syracuse, NY 13210, USA

[2] US Forest Service, Northern Research Station, Durham, NH 03824, USA

[3]University of Alaska Anchorage, Biological Sciences Department, AK 99508, USA

[4]Cary Institute of Ecosystem Studies, Millbrook, NY 12545, USA and Department of Ecology and Evolutionary Biology, University of Connecticut, Storrs, CT 06269, USA

*tpuntsag@syr.edu

## Supplementary table and figures

**Table S1**: Climate oscillation indices and their correspondence to weighted annual precipitation isotopes at the HBEF.

| Index | by Variable | Bivariate $r^2$ (P value) | Kendall Tau b (Prob>\|Tau b\|) | Spearman Rho (Prob>\|Rho\|) | Hoeffding D (Prob>D) |
|---|---|---|---|---|---|
| AMO | $\delta^{18}O$ | **0.69 (<.0001)** | **-0.71 (<.0001)** | **-0.89 (<.0001)** | **0.42 (<.0001)** |
| | *d- excess* value | **0.83 (<.0001)** | **0.77 (<.0001)** | **0.94 (<.0001)** | **0.53 (<.0001)** |
| | $\delta^2H$ | **0.3 (0.0004)** | **-0.43 (<.0001)** | **-0.60 (<.0001)** | **0.12 (<.0001)** |
| NAO | $\delta^{18}O$ | 0.02 (0.35) | 0.12 (0.254) | 0.18 (0.2517) | **0.03 (0.0274)** |
| | *d- excess* value | 0.035 (0.2274) | -0.12 (0.2718) | -0.18 (0.26) | 0.01 (0.1486) |
| | $\delta^2H$ | 0.014 (0.7) | 0.04 (0.6832) | 0.08 (0.631) | 0.00 (0.3688) |
| PNA | $\delta^{18}O$ | 0.014 (0.44) | -0.06 (0.5578) | -0.10 (0.5439) | 0.00 (0.4184) |
| | *d-excess* value | 0.008 (0.57) | 0.004 (0.9666) | 0.02 (0.9185) | 0.00 (0.4109) |
| | $\delta^2H$ | 0.014 (0.44) | -0.04 (0.6908) | -0.05 (0.7292) | 0.00 (0.3089) |
| AO | $\delta^{18}O$ | 0.0001 (0.94) | -0.04 (0.6832) | -0.08 (0.5931) | 0.00 (0.4782) |
| | *d- excess* value | 0.00085 (0.85) | 0.09 (0.4084) | 0.13 (0.4065) | -0.01 (0.8108) |
| | $\delta^2H$ | 0.0001 (0.93) | -0.04 (0.7298) | -0.08 (0.6018) | 0.00 (0.4111) |

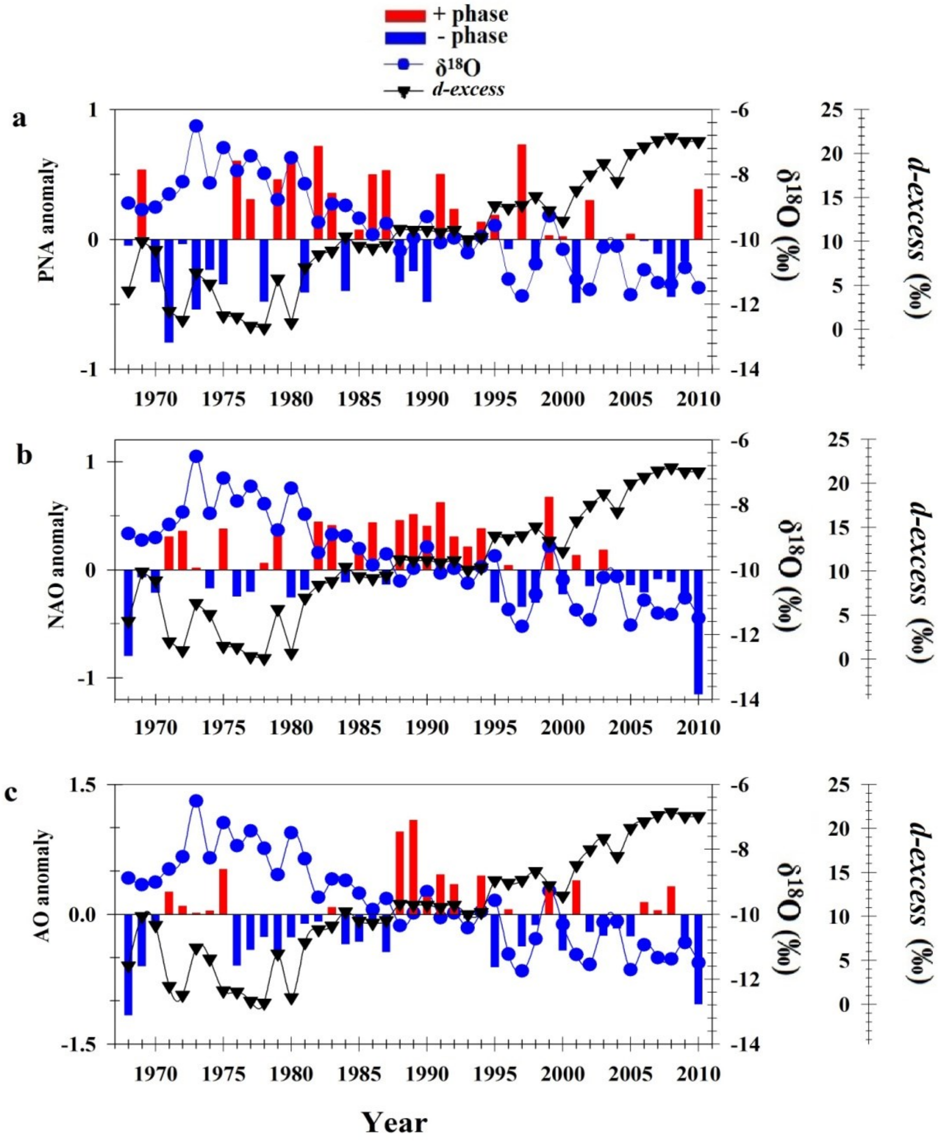


**Figure S2**: **HBEF precipitation water isotopes and *d-excess* value response during different climate oscillations throughout the study period.** (a) averaged monthly to annual, PNA anomalies based on modified pointwise method http://www.cpc.ncep.noaa.gov/products/precip/CWlink/pna/nao.shtml (b) monthly mean to annual , NAO index since January 1950 same date source of PNA. (c) monthly mean to annual AO at around 55$^{o}$N latitude( http://www.ncdc.noaa.gov/teleconnections/ao/).

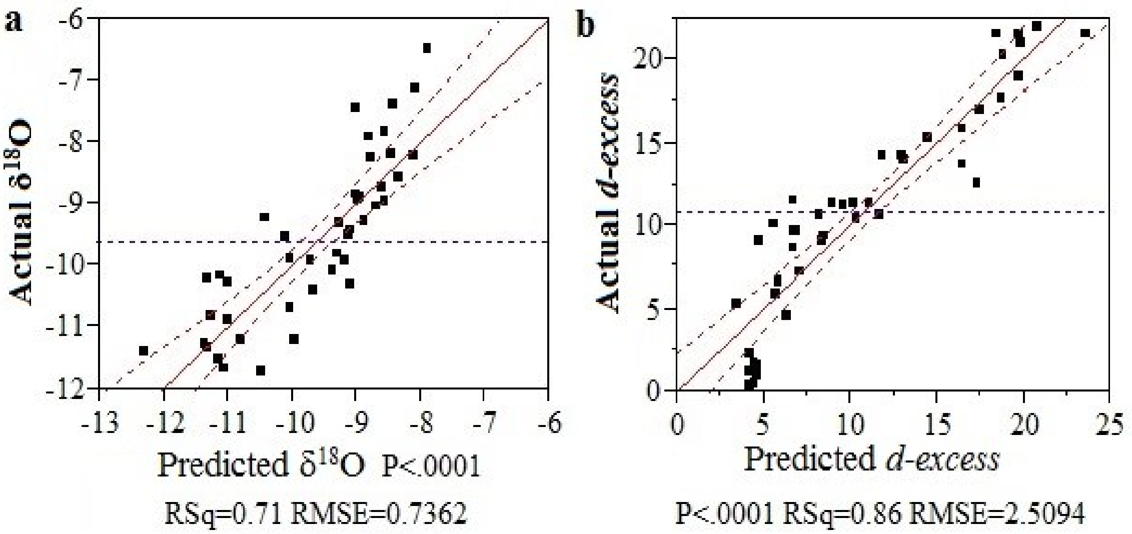


**Figure S3**: **Plot of predicted vs measured $\delta^{18}$O and *d- excess* values.** The $\delta^{18}$O plot based on the step 2 equation relating $\delta^{18}$O values to AMO and surface air temperature. The *d-excess* plot is based on the step 4 equation relating *d-excess* to AMO, precipitation and AO. The equation explains 70% and 84% of the variability in measured precipitation $\delta^{18}$O and *d-excess* values, respectively.

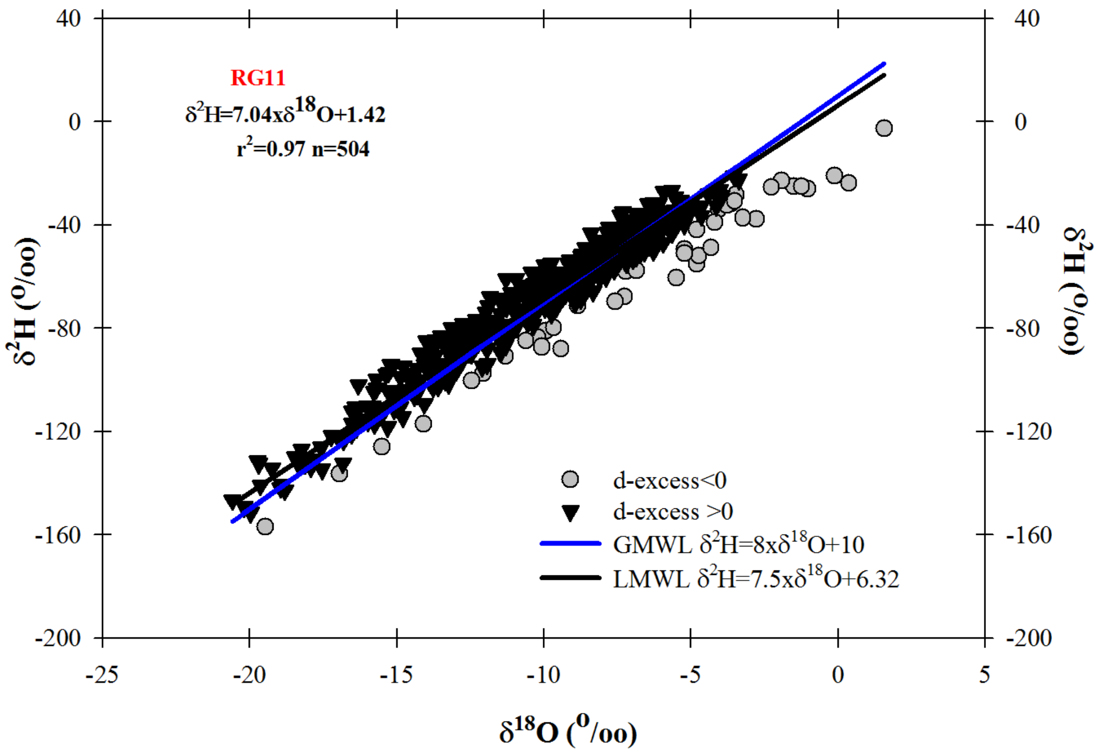


**Figure S4**: **A lines represent the Ottawa, Ontario line (LMWL) and global meteoric line (GMWL). Monthly δ values for RG 11.** The HBEF samples may be exhibiting some evaporative effects and hence were skewed slightly as indicated by the respective slopes of the HBEF samples (7.0) versus the Global (8.0) and 7.5 for the Ottawa precipitation samples.
